# Review of canine dilated cardiomyopathy in the wake of diet-associated concerns

**DOI:** 10.1093/jas/skaa155

**Published:** 2020-06-15

**Authors:** Sydney R McCauley, Stephanie D Clark, Bradley W Quest, Renee M Streeter, Eva M Oxford

**Affiliations:** 1 BSM Partners, Bentonville, AR; 2 The Heart Vet, Ithaca, NY

**Keywords:** carnitine, deficiency, diet, dilated cardiomyopathy, dog, taurine

## Abstract

Dilated cardiomyopathy (DCM) has been in the literature and news because of the recent opinion-based journal articles and public releases by regulatory agencies. DCM is commonly associated with a genetic predisposition in certain dog breeds and can also occur secondary to other diseases and nutritional deficiencies. Recent communications in veterinary journals have discussed a potential relationship between grain-free and/or novel protein diets to DCM, citing a subjective increase in DCM in dog breeds that are not known to have a genetic predisposition for the disease. This literature review describes clinical presentations of DCM, common sequelae, treatment and preventative measures, histopathologic features, and a discussion of the varied etiological origins of the disease. In addition, current literature limitations are addressed, in order to ascertain multiple variables leading to the development of DCM. Future studies are needed to evaluate one variable at a time and to minimize confounding variables and speculation. Furthermore, to prevent sampling bias with the current FDA reports, the veterinary community should be asked to provide information for all cases of DCM in dogs. This should include cases during the same time period, regardless of the practitioner’s proposed etiology, due to no definitive association between diets with specific characteristics, such as, but not limited to, grain-free diets and those containing legumes, novel protein diets, and those produced by small manufacturers to DCM in dogs. In summary, in order to determine if certain ingredients, categories of diets, or manufacturing processes are related to an increased risk of DCM, further studies investigating these variables are necessary.

## Introduction

Historically, dilated cardiomyopathy (**DCM**) has been considered to be primarily an inherited disease, with higher prevalence in specific dog breeds (Dukes-McEwan et al., 2003; Sammarco, 2008). However, other causes of DCM include specific nutrient deficiencies (Freeman et al., 2001; Backus et al., 2003) and concurrent diseases, such as endocrine (Karlapudi et al., 2012; Janus et al., 2014), myocarditis, and chronic tachycardia (Calvert et al., 1997). Recently, there has been concern regarding the relationship of diets with specific characteristics, such as, but not limited to, grain-free diets and those containing legumes, novel protein diets, and those produced by small manufacturers to DCM in dogs (Freeman et al., 2018); however, no definitive link has been established at this time.

In order to better understand the disease process, a knowledge of the incidence, clinical manifestations, diagnostics, and potential treatments is required. Research studies must be carried out evaluating one variable at a time, while mitigating sampling bias of data collected, to effectively identify potential new causes of DCM (Simundić, 2013). Additionally, robust statistical designs that look at the effect of single dietary components, in controlled environments, with robust outcomes related to metabolism will allow for an understanding of potential disease etiology. This review covers the various etiologies known to cause DCM in dogs, including genetics, hypothyroidism, myocarditis, chronic tachycardia, and certain known dietary deficiencies (Phillips and Harkin, 2003; Sanderson, 2006; Wess et al., 2010b; Beier et al., 2015; Hallman et al., 2019; Vollmar et al., 2019). It also includes other potential diet-associated etiologies that have been recently suggested (Santilli et al., 2017).

## Incidence

Information regarding the incidence of DCM in the dog population is limited. Up to 75% of all cardiovascular disease in the dog is chronic degenerative valve disease (Kvart and Häggström, 2000; Kahn, 2005). The second most common heart disease (and most common primary myocardial disease) in the dog is reported to be DCM. According to the Veterinary Medical Database (Sisson et al., 2000) from 1986 to 1991, 0.5% of the dogs evaluated at U.S. referral-hospitals were diagnosed with DCM. Moreover, a U.S. veterinary teaching hospital study that included all dogs evaluated in that hospital between 1995 and 2010 (90,004) reported a DCM incidence rate of 0.4%. Interestingly, a subset of dogs from the same study that only included dogs with an inherited disease (27,254 cases) observed an incidence rate of 1.3% in this specific group (Bellumori et al., 2013). In a European study, 1.1% of the dog population seen at veterinary hospitals were diagnosed with DCM (Fioretti and Delli, 1988). It can be hypothesized that the occurrence of DCM may be different in the general dog population since dogs presented to veterinary hospitals (including referral institutions) are only a subset and may not be representative of the overall dog population (Redfield et al., 1993). If one considers the estimated total number of dogs in the United States equals 77,000,000 (AVMA, 2019), the published incidence studies (Fioretti and Delli, 1988; Sisson et al., 2000) suggest that a minimum of 308,000 to 1,001,000 dogs in the United States have DCM at any given time. In June 2019, the Food and Drug Administration (**FDA**) released a public statement that 560 dogs were reported with potential diet-related DCM (FDA, 2019a). If the report was accurate, these 560 cases would represent 0.05% to 0.1% of dogs in the United States with DCM.

DCM has historically been an inherited, genetically linked condition. Published surveys, in North America, reported a higher incidence of DCM in Doberman Pinschers, Irish Wolfhounds, Great Danes, Boxers, and American Cocker Spaniels (Dukes-McEwan et al., 2003). Other breeds, such as Bulldogs (Meurs, 2003), Golden Retrievers, and Saint Bernards, have also been reported to have a higher incidence of DCM (Backus et al., 2003, 2006; Fascetti et al., 2003; Belanger et al., 2005; Vollmar et al., 2013). Additionally, European sources reported a higher incidence in Airedale Terriers, Doberman Pinschers, Newfoundlands, Scottish Deerhounds, and English Cocker Spaniels (Meurs, 2010). Typically, DCM in dogs is more prevalent in males than in females (Oyama, 2015). More specifically, Doberman Pinschers have a reported incidence rate of 50% in males and 33% in females (Oyama, 2015), while Irish Wolfhounds have an overall breed incidence of 25%. DCM is also a disease of middle aged to older dogs (Oyama, 2015); there are cases of juvenile DCM reported in Portuguese Water Dog puppies (Sleeper et al., 2002).

The incidence of DCM resulting from other disease states (hypothyroid disease, myocarditis, chronic tachycardia) is poorly understood. DCM related to low taurine levels has been noted in Cocker Spaniels (Kittleson et al., 1997; Egenvall et al., 2006), Golden Retrievers (Belanger et al., 2005; Kaplan et al., 2018), and foxes (Moise et al., 1991), though the incidence is unknown.

## Clinical Manifestation

Due to the compensatory mechanisms of the cardiovascular system, DCM initially manifests in a subclinical phase, occult phase, which has been described in detail in Doberman Pinschers (Calvert et al., 1997). Decreased systolic function and arrhythmias may be present in the occult phase; however, no clinical signs may be observed. Therefore, early screening in at-risk breeds is important (Vollmar, 1999). Throughout the natural course of the disease progression, cardiac contractile function diminishes, resulting in decreased cardiac output. Furthermore, continued myocardial remodeling may result in dangerous arrhythmias. The tipping point in the disease process is when clinical signs, such as exercise intolerance, congestive heart failure (**CHF**), syncopal episodes, and sudden cardiac death (**SCD**), occur (O’Grady and Horne, 1992). Often, SCD may be the first manifestation of this disease and can occur in up to 40% of Doberman Pinschers (Koch et al., 1996).

## Histopathological Manifestation

Histopathological changes vary from myocardial samples in dogs with DCM, reflecting the numerous underlying etiologies (Tidholm et al., 2001; Janus et al., 2014). Some samples may have wavy, attenuated myofibers, while others have interstitial fibrosis and myofibrillar degeneration. Other samples exhibit more severe replacement fibrosis, myocytolysis, and fatty infiltration (Tidholm et al., 1997); the latter of which may be associated with end-stage arrhythmogenic right ventricular cardiomyopathy (Basso et al., 2004).

## Diagnostic Tools

### Physical examination

During the occult phase of DCM, physical examination of the cardiovascular system may be normal, but physical exam findings can reveal jugular pulses, an irregular heart rhythm, weak pulses, pulse deficits, or a systolic murmur with a point of maximum intensity in the left sixth intercostal space (Guglielmini, 2003; Kahn, 2005). If CHF is present, crackles or muffled heart and lung sounds may be auscultated (Sisson et al., 2000).

### Imaging modalities

Thoracic radiographs can reveal generalized enlargement of the cardiac silhouette or more specifically enlargement in the area of the left atrium and left ventricle. In addition, distention of the pulmonary veins, tracheal elevation, and pulmonary edema with or without pleural effusion or pleural fissure lines may also be present (Sisson et al., 2000).

Echocardiography is necessary to definitively differentiate DCM from other cardiovascular diseases (Bonagura and Herring, 1985). Classic findings include increased end-diastolic and end-systolic diameter of the left ventricle (Sisson et al., 2000) with concurrent decreased left ventricular ejection fraction and fractional shortening (Martin et al., 2009).

### Electrocardiogram

Electrocardiogram (**ECG**) evaluation can reveal supraventricular and ventricular arrhythmias, in addition to wide and tall P (indicative of atrial enlargement) and R (indicative of ventricular enlargement) waves (Meurs, 2003). It is important to note that a normal ECG does not rule out the presence of DCM, as most arrhythmias are intermittent and have high day-to-day variability (Spier and Meurs, 2004).

### Twenty-four-hour Holter monitoring

Holter monitoring is the gold standard screening tool to detect arrhythmias in dogs, allowing for detection of over a 24-h window. The presence of greater than 50 ventricular premature complexes of a Holter monitor is indicative of DCM in Doberman Pinschers (Singletary et al., 2012).

### Cardiac biomarkers

Diagnostic tools are commonly coupled with assessing cardiac biomarkers for a more comprehensive picture of the DCM stage being evaluated. Measurement of serum concentrations of the N-terminal end of the brain natriuretic peptide (**NT-proBNP**) offers a measure of chronic, abnormal stretch, or strain on the myocardium. This has been shown to be a sensitive and specific marker of underlying cardiac diseases (Boswood et al., 2008; Oyama and Singletary, 2010; Singletary et al., 2012) and, in general, the higher the NT-proBNP, the more severe the cardiac disease (Anjos et al., 2015). For example, NT-proBNP levels of less than 800 pmol/L are expected in a dog with no cardiac disease, while the levels of >2,700 pmol/L may be appreciated in a dog with active CHF (Anjos et al., 2015). When used in conjunction with 24-h Holter monitoring, NT-proBNP levels can help to detect occult phase DCM (Kluser et al., 2016). However, excretion of NT-proBNP occurs exclusively through the kidneys and so the renal function must be closely evaluated when using NT-proBNP as a biomarker (Schmidt et al., 2009).

Measurement of cardiac troponin-I (**cTnI**) concentration is a beneficial biomarker that indicates acute myocardial damage (Langhorn and Willesen, 2016). Troponin, an integral protein of the contractile apparatus, is released from cardiomyocytes during death and has a volatile half-life of approximately 2 h (Dunn et al., 2011). The concentration of cTn has been shown to increase proportionally to the degree of myocardial damage (Barison et al., 2010) and though cTnI concentration may indicate the degree of myocardial damage, this concentration does not differentiate between the causes of myocardial disease. Interestingly, in one study comparing Doberman Pinschers with and without DCM, it was observed that cTnI was significantly elevated in the group with DCM (Wess et al., 2010a).

### Differential diagnosis on imaging studies

Chronic valve disease is the most common heart disease in dogs (Kvart and Häggström, 2000; Kahn, 2005; Freeman, 2010). Atrioventricular valve disease, especially mitral (left-sided) valve disease, can have many characteristics that appear similar to DCM on radiographs and echocardiograms (Carr, 2017). These include a dilated left atrium and ventricle with the overall heart silhouette resembling cardiomegaly as the disease progresses (Prosek et al., 2007; Janus et al., 2014). Right-sided valve (tricuspid) disease can cause an increase in right atrium size, due to increased tricuspid regurgitation. It can result in right ventricular hypertrophy from cardiac compensation, which can lead to CHF, increasing the overall size of the heart silhouette (Favril et al., 2018).

Accumulation of fluid in the pericardial sac covering the heart, pericardial effusion, can be a symptom of heart disease in dogs. Additionally, neoplasia of the heart, CHF, pericarditis, and left atrial rupture can cause pericardial effusion (Olcott and Sleeper, 2010). Pericardial effusion can cause the heart silhouette to look enlarged on radiographs and be mistaken for other diseases, such as DCM, which also makes the cardiac silhouette appears large (Jutkowitz, 2008).

## Prevention of DCM

Breeding recommendations to help minimize the risk of DCM are often described through breed-specific organizations. Selective breeding, in breeds known to have an inherited predisposition to the disease, can be a measure of DCM prevention. Prevention of non-inherited cases of DCM is difficult in most cases, as arrhythmias, infectious disease, and hypothyroidism are not preventable. Feeding a diet with proper essential nutrient concentrations is within reach for most pet owners; however, it should be considered that there are potentially breed-specific requirements for some amino acids, such as taurine (Ko et al., 2007; Freeman et al., 2018; Kaplan et al., 2018). Further breed-specific studies are warranted, and avoiding nutritional deficiencies is imperative especially in breeds that may need higher concentrations of taurine or l-carnitine, such as Cocker Spaniels and Golden Retrievers (Kittleson et al., 1997; Kaplan et al., 2018).

## Treatment of DCM

### Treatment of occult DCM

Early screening and detection of DCM are the keys to prolonging life in dogs. Once diagnosed with DCM, the administration of pimobendan, a positive inodilator, at 0.25 mg/kg by mouth, every 12 h, significantly delays the onset of CHF in Doberman Pinschers (Summerfield et al., 2012) and other breeds (Boswood et al., 2018). Therefore, pimobendan has become a standard therapy in dogs with decreased systolic function and left ventricle dilation.

### Core treatment for CHF secondary to DCM

Treatment of CHF, for dogs diagnosed with DCM, is similar to other CHF etiologies: decreasing preload and afterload while maintaining systemic blood pressure. In most cases, triple therapy is warranted for treating CHF. Triple therapy includes prescribing pimobendan (0.25 mg/kg by mouth, twice daily), a diuretic such as furosemide (typical starting dose is 2 mg/kg by mouth, twice daily), and an angiotensin-converting enzyme (ACE) inhibitor such as enalapril (0.25 to 0.5 mg/kg by mouth, twice daily; Plumb, 2018). Care must be taken when administering furosemide, due to the risk of severely decreasing the preload, as dogs with severely compromised systolic function cannot easily maintain normal blood pressure. It is recommended that a Doppler blood pressure be evaluated in any dog with reported lethargy after initiating diuretic therapy, in order to screen for hypotension secondary to poor cardiac output.

### Holter monitoring for arrhythmias secondary to DCM

Dogs diagnosed with DCM should have a 24-h Holter monitor placed, regardless of clinical status (Spier and Meurs, 2004). The threshold for treatment of arrhythmias, noted on Holter monitoring, is complex and varies due to clinician experience and preference. Furthermore, anti-arrhythmic pharmaceuticals have the potential to be pro-arrhythmic. Thus, a baseline Holter recording is important before the initiation of any medication. It is estimated that SCD (presumptively secondary to ventricular arrhythmias) may occur in up to 40% of Doberman Pinschers with DCM (Koch et al., 1996; Oyama, 2015). While no anti-arrhythmic therapy can prevent the occurrence of SCD, appropriate therapy may reduce the risk.

### Additional therapies for specific DCM etiologies

Early treatment of DCM is dependent on the underlying etiology. Taurine and l-carnitine supplementation may help improve clinical signs and echocardiographic parameters (Kittleson et al., 1997; FDA, 2019a). In dogs with known or suspected nutritional deficiencies, secondary to inappropriate diet or genetic predisposition, taurine concentrations, and carnitine concentrations should be assessed and supplementation initiated. Taurine concentrations can be assessed in whole blood. Taurine supplement dose recommendations are 500 mg, by mouth, every 8 to 12 h (Kittleson et al., 1997). It is recommended that supplements should be given with food. While supplementation would ideally be initiated only if found to be deficient based on diagnostics, because supplementation is safe, supplementation can be initiated if deficiency is a suspected concern (Sanderson, 2006). Supplementation without diagnostics may be indicated when owners decline these diagnostics due to the expense of testing.

Free carnitine, total carnitine, and carnitine ester concentrations can be assessed via plasma or urine. Carnitine concentrations can also be assessed via skeletal or cardiac muscle biopsies. The appropriate effective supplementation dose may depend on the type of deficiency. The commonly recommended dose of 50 to 100 mg/kg, every 8 h, may be appropriate for the management of systemic deficiency (Keene, 1991; Sanderson, 2006). Myocardial deficiency, however, may require a higher dosage of 200 mg/kg, by mouth, to maximize the chances that carnitine supplementation will improve cardiac function, based on a limited number of cases from the University of Minnesota (Sanderson, 2006). The usage of l-carnitine for supplementation is critical since d-carnitine can interfere with l-carnitine utilization (Pion et al., 1998). Additionally, there are several forms of l-carnitine, including l-carnitine esters (i.e., acetyl- and lauroyl-) and l-carnitine salts (i.e., -tartrate, -fumarate, -magnesium citrate, and propionyl-). The bioavailability of these forms can vary. For example, one study observed in piglets that l-carnitine salts have a similar bioavailability to free l-carnitine (Eder et al., 2005). l-Carnitine esters were reported to have the lowest bioavailability in comparison to the other forms. Additionally, l-carnitine tartrate was absorbed faster than other carnitine compounds. However, propionyl-l-carnitine was not assessed and has been shown to have an affinity for cardiac muscle.

Multiple studies have examined the effects of propionyl-l-carnitine on cardiac metabolism (Siliprandi et al., 1991; Di Lisa et al., 1994; Schonekess et al., 1995; Mingorance et al., 2011). In particular, supplementation of propionyl-l-carnitine may be beneficial in cardiac disease since it has a high affinity for cardiac muscle (Mingorance et al., 2011). In cardiac muscle, propionyl-l-carnitine is converted to free l-carnitine and propionyl coenzyme A, which plays a crucial role in the metabolism of carbohydrates and lipids leading to an improvement in ATP efflux. Additionally, it stimulates the tricarboxylic acid cycle through succinate synthesis, which can help protect against ischemia (Siliprandi et al., 1991; Mingorance et al., 2011). Therefore, propionyl-l-carnitine may be the more beneficial form of supplementation for the management of cardiac disease, although its effects compared with other forms have yet to be studied in dogs.

## Etiology

**Figure 1.**
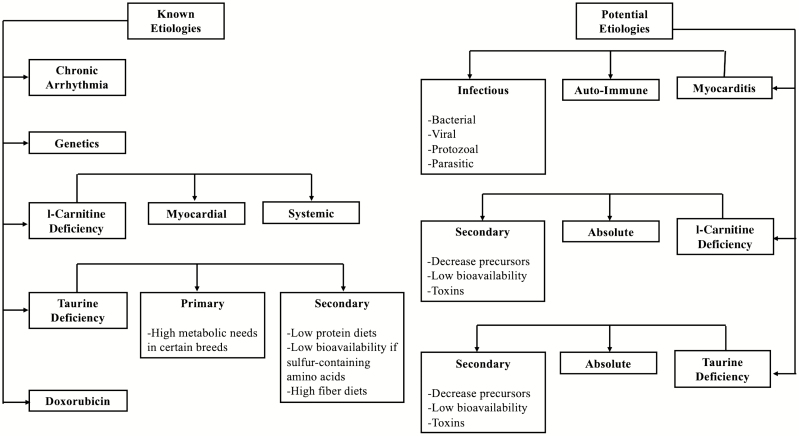
Known and potential etiologies associated with DCM.

Multiple etiologies of the DCM phenotype exist, with the most commonly diagnosed etiology being the presumptively inherited DCM of certain dog breeds (Stern and Ueda, 2019). Other etiologies of DCM are reported and include infectious/inflammatory insults (myocarditis), endocrine disease, high arrhythmic load, toxins, and nutritional deficiencies (Figure 1). However, the definitive etiology in many cases is limited due to the difficulty of antemortem testing of the myocardium and out-of-pocket cost to screen for nutritional deficiencies and infectious agents.

### Inherited DCM

DCM is a naturally occurring heritable disease in certain dog breeds, and genetics remains the most commonly attributed cause in the dog (Stern and Ueda, 2019). The genetic predisposition of the Doberman Pinscher to DCM has been investigated in detail (Wess et al., 2010a; O’Sullivan et al., 2011; Meurs et al., 2012; Steudemann et al., 2013; Sosa et al., 2016). Inheritance of DCM in other breeds has also been considered (Kittleson et al., 1997; Dambach et al., 1999; Vollmar, 2000; Werner et al., 2008; Simpson et al., 2016), though causative genetic mutations are not always identified (Sammarco, 2008). Genes thought to be associated with DCM are dystrophin (*DMD*) in German Shorthaired Pointers, striatin (*STRN*) in Boxers, and both pyruvate dehydrogenase kinase 4 (*PDK4*) and a locus on chromosome 5 in Doberman Pinschers (Sammarco, 2008). These mutations are proposed to influence cellular energy production, cell signaling, and cellular communication, respectively.

Inheritance may explain the increased prevalence of disease in certain breeds; however, many dog breeds develop DCM without a known genetic predisposition, and, therefore, causative factors other than genetics must be considered (Sammarco, 2008; Meurs, 2010; O’Sullivan et al., 2011). Additionally, it is possible for DCM to be diagnosed in mixed-breeds dogs that are genetically linked to DCM-predisposed breeds. In a study by Donner et al. (2019), evaluating 100,000 purebred and mixed-breed dogs, it was noted that out of 152 genetic-related diseases tested, 2 out of 100 mixed-breed dogs and 5 out of 100 purebred dogs were at risk to develop a genetically related disease. In addition, 40 out of 100 purebreds and 28 out of 100 mixed-breeds were carriers for at least one genetically linked disease. Thus, mixed-breed dogs can be predisposed to developing genetically linked disease phenotypes, similar to their purebred counterparts (Donner et al., 2019).

Bellumori et al. (2013) evaluated records of 27,254 dogs with an inherited disease seen at a U.S. veterinary teaching hospital (Bellumori et al., 2013). In this report, 361 dogs were diagnosed with DCM, and, of those, 32 (8.9%) were mixed breed dogs (Bellumori et al., 2013). Extrapolating from this, 8.9% of dogs with an inherited disease had DCM and were classified as mixed breed dogs in this retrospective study. Thus, more research into the genetic components of DCM in purebred and mixed breed dogs are warranted.

### Myocarditis

DCM and subsequent CHF are the most common clinical sequelae to myocarditis in humans and dogs (Cooper, 2009; Ford et al., 2017; Santilli et al., 2017). Myocarditis in humans and dogs is histopathologically defined by the presence of an inflammatory infiltrate, with or without myocyte necrosis, within the myocardium (Cooper, 2009; Ford et al., 2017). While the diagnosis of myocarditis via endomyocardial biopsy is the standard of care in humans (Cooper, 2009), it is less commonly performed in dogs. Most definitive myocarditis diagnoses in dogs are obtained through postmortem histopathology. Due to limitations in definitive testing modalities in dogs, myocarditis is suspected to be underrepresented as a cause of DCM. Myocarditis in humans and dogs is commonly viral in origin, with parvovirus being frequently reported in dogs (Meunier et al., 1984; Ford et al., 2017). Myocarditis is also reported in cases infected with *Borrelia burgdorferi* (Janus et al., 2014; Detmer et al., 2016) caused by bacteria, including *Bartonella* (Santilli et al., 2017), *Trypanosoma cruzi* (Williams et al., 1977), and *Neospora caninum* (Barber and Trees, 1996). In many cases of suspected myocarditis, the source of infection is not identified. However, certain infectious organisms are known to be present in greater prevalence in different geographic locations. This may lead to an increased risk of myocarditis cohorts, depending on geographical location. For example, *T. cruzi* infections occur most commonly in the south (Hodo et al., 2019), while exposure to *B. burgdorferi* (Lyme disease) is most prevalent within the United States in the northeast and northern Midwest (Watson et al., 2017). Furthermore, dogs with extensive travel and outdoor exposure may have an increased risk of myocarditis. In one extreme example, military working dogs have a higher risk of developing endocarditis and myocarditis than the general U.S. canine population due to increased exposure (Shelnutt et al., 2017; Davis et al., 2020).

### Hypothyroid disease

Thyroid hormones regulate key proteins involved in positive cardiac ionotropy and chronotropy (Scott-Moncrieff, 2012). Clinically hypothyroid dogs may have decreased systolic function, low QRS voltages, weak apex beat, and sinus bradycardia (Panciera, 1994b, 2001; Scott-Moncrieff, 2012) due to poor hormone regulation. This is further supported in hypothyroid dogs treated pharmacologically for hypothyroidism (Taylor et al., 1969; Panciera, 1994a, 1994b). Dogs were observed to have an improved cardiac function with decreases in left ventricular diameter, increases in ejection fraction, as well as increases in fractional shortening. Thus, hypothyroidism can lead secondarily to DCM.

### Tachycardia-induced cardiomyopathy

Heart rate directly affects left ventricular systolic function. Experimentally induced tachycardia (>200 beats/ min) can create a DCM phenotype with concurrent CHF and reversible decreased systolic function in dogs (Armstrong et al., 1986; O’Brien et al., 1990). Additionally, reversible myocardial dysfunction in humans and dogs has been reported in cases of naturally occurring tachycardia (Grogan et al., 1992; Wright et al., 1999). The pathophysiological mechanisms resulting in tachycardia-induced cardiomyopathy are nonspecific and similar to those noted with other forms of DCM phenotype (O’Brien et al., 1990). These mechanisms include increased oxygen demand, decreased myocardial blood supply (Spinale et al., 1992), loss and eccentric hypertrophy of myocytes (Kajstura et al., 1995; Jovanovic et al., 1999), and abnormal calcium handling and reduced adenosine triphosphate production (Perreault et al., 1992).

It is important to emphasize that while there are numerous known causes of DCM in the dog, often, due to the limitations in testing and financial constraints of the owner, the definitive cause of a DCM phenotype is not determined. However, various tests, such as 24-h Holter monitoring, cTnI concentration, infectious disease testing, thyroid hormone levels, and plasma taurine (discussed below), can be useful in narrowing down the differential list. In some fortunate cases, a definitive cause of DCM can be identified, and cardiac function may improve with treatment of the underlying disease (e.g., severe hypothyroid disease). Often an underlying disease is not identified despite exhaustive testing, and these cases of DCM will continue to progress over time.

## Diet-associated DCM

### Diets low in protein, taurine, and/or taurine precursors

Diets low in protein, taurine, and sulfur-containing amino acid precursors have been associated with taurine-deficient DCM. Low protein diets designed for the management of urate stones were noted to be associated with DCM. This may be due to low protein diets being low in essential and nonessential amino acids or vital precursors for carnitine and taurine synthesis. Although this report was not a controlled study, when these diets were supplemented with taurine and l-carnitine, DCM clinical signs were reversed and dogs lived longer (Sanderson et al., 2001). In a 2006 study, two unrelated dogs on a tofu-based diet had a taurine deficiency. While the diet was lower in protein, AAFCO requirements for protein were met. Tofu is made from soybean-cure, which is low in sulfur-containing amino acids and devoid of taurine and may have contributed to the cause (Spitze et al., 2003).

### Fiber

Dietary fiber is a functional component in pet food as it affects stool quality, can assist with satiety in weight-loss diets, and moderate glucose absorption for diabetic animals. Additionally, plant polysaccharides can be metabolized by microbes and produce short-chain fatty acids, which can be used for energy. Moderate levels of dietary fiber can improve gut health and support the balance of the gut microbiota. In a 2010 study, it was noted that 7.5% of beet pulp was enough dietary fiber to improve the diversity of the fecal microbiome, by providing fermentable substrates for the microbes, without causing adverse changes to nutrient absorption (Middelbos et al., 2010). This is important because the gastrointestinal tract has been identified, in pig models, as a primary location where sulfur-containing amino acids are metabolized (Bauchart-Thevret et al., 2009). Interestingly, intestinal metabolism of sulfur-containing amino acids, such as methionine and cysteine, can greatly affect the concentration in the plasma. Furthermore, these amino acids have been documented to have the potential to support epithelial cells and gut function (Bauchart-Thevret et al., 2009).

However, diets high in fiber, more specifically insoluble fiber, can decrease the crude protein digestibility in the hindgut (Wilfart et al., 2007). In pigs, it has been demonstrated that certain dietary fiber, such as non-starch polysaccharides, are relatively nonfermentable and have anti-nutritive effects (Cadogan and Choct, 2015). This can lead to a decrease in sulfur-containing amino acids and result in nutrient deficiencies, such as taurine or carnitine. Taurine, carnitine, and other nutrients can be indirectly affected due to the potential decrease in protein digestibility. These nutrients are vital in cardiac muscle function. This was observed in medium and large breed dogs given beet pulp (Ko and Fascetti, 2016). The study hypothesized that diets, which were formulated to meet AAFCO requirements, may not actually be meeting the dogs’ nutritional needs due to fiber’s negative effect on nutrient absorption (Ko and Fascetti, 2016). Fiber can also influence fermentation byproducts from microbes in the hindgut and hinder reabsorption of taurine, even if taurine is biosynthesized in sufficient amounts (Ko and Fascetti, 2016). Furthermore, fiber can affect nutrient absorption in multiple ways. Fiber can delay gastric emptying, which can slow down absorption and affect the location where nutrients are normally absorbed. Soluble fibers can create a gel around carbohydrates, which can lead to indirect effects on blood glucose concentrations. For example, the fiber in the form of unrefined cereals, legumes, nuts, oilseeds, fruits, and vegetables can reduce the absorption of fats and fat-soluble vitamins, such as Vitamin A and carotenoids (Gibson, 2007). Benefits and negative effects of dietary fiber should be considered when formulating diets.

## Nutrient Deficiencies

Deficiencies in certain nutrients are known to play a role in the development of DCM. These include taurine, carnitine, and their precursors, such as methionine and cysteine (taurine) and lysine and methionine (carnitine). Thiamine, copper, potassium, vitamin E, and selenium deficiencies have also been associated with myocardial damage; however, further investigation is needed to determine if they are contributing factors to the development of DCM.

### Potassium

A deficiency in potassium can lead to the development of cardiovascular disease in mammals (Dow et al., 1992; Kjeldsen, 2010). Dow et al. (1992) observed that decreased potassium intake can induce a taurine depletion that can contribute to cardiovascular diseases in cats (Dow et al., 1992). Cats were fed diets containing 0.2% potassium on a dry matter basis. Although the diets had enough dietary taurine at 0.12%, on a dry matter basis, three out of six cats developed cardiomyopathy and thromboembolism. However, cats fed a potassium-replete diet did not develop a taurine deficiency (Dow et al., 1992). The study concluded an association between taurine and potassium balance in cats, suggesting taurine status and cardiovascular diseases may be associated to a concurrent potassium depletion.

Additionally, in a study evaluating 238 dogs with low sodium to potassium ratio (Nielsen et al., 2008), 21 dogs had concurrent cardiovascular disease, more specifically, DCM. However, due to the lack of controlled variables, it was difficult to determine if a low sodium to potassium ratio increases the risk of cardiovascular disease or is a symptom of the disease. Given the current literature, further studies should be conducted to determine the mechanism behind potassium deficiency and the development of cardiovascular disease and taurine deficiency.

### Choline

Choline is a constituent of the neurotransmitter acetylcholine as well as a component of cell and mitochondrial membranes. Due to its abundance in tissues, choline influences many processes in the body, such as lipid metabolism and signaling (da Costa et al., 1995; Zeisel, 2006), one-carbon metabolism (Niculescu et al., 2004, 2006; Mehedint et al., 2010), intestinal circulation of bile and cholesterol (Dawson et al., 2009), and mitochondrial bioenergetics (Teodoro et al., 2008). Choline has two major fates, though, one is to be phosphorylated and used to make phospholipids and the other is to be oxidized and used as a donor of methyl groups. As a methyl donor, choline is important for the regeneration of methionine from homocysteine (Wong and Thompson, 1972; Mudd and Poole, 1975; Finkelstein et al., 1983). This reaction is catalyzed by betaine homocysteine methyltransferase, where betaine, a metabolite of choline, serves as the methyl donor (Finkelstein et al., 1983). When choline stores are deficient, the capacity to methylate homocysteine to methionine is diminished resulting in increased plasma homocysteine concentration (da Costa et al., 2005). Elevated homocysteine concentrations are associated with increased risk of cardiovascular diseases in humans (Gerhard and Duell, 1999). Hyperhomocysteinemia adversely affects myocardial compliance and contractility of the heart (Joseph et al., 2003; da Costa et al., 2005; Herrmann et al., 2007). Dietary supplementation of choline and betaine on cardiovascular disease has been evaluated in humans (Schwab et al., 2002; Olthof et al., 2003, 2005; Steenge et al., 2003). However, results on whether the supplementation is beneficial or deleterious to cardiac disease are conflicting. For example, one study showed that the levels of choline intake in humans were inversely associated with homocysteine concentrations in the blood (Dalmeijer et al., 2008). However, a follow-up study of the same cohort failed to show a difference in cardiovascular risk on varying dietary choline intake (Bidulescu et al., 2007). In addition, choline is involved in the production of trimethylamine N-oxide (**TMAO**), in high concentrations in the serum, promotes inflammation, and can lead to the development of cardiovascular diseases in humans (Randrianarisoa et al., 2016). TMAO is the oxidized product of trimethylamine. Trimethylamine is produced from bacteria strains, such as Firmicutes, Actinobacteria, and Proteobacteria, in the presence of choline degradation in the gastrointestinal tract (Fennema et al., 2016). This product is found to be higher in dogs and humans with CHF, secondary to degenerative valve disease. Of the dogs in the FDA’s June 2019 report diagnosed with DCM, 15% (FDA, 2019a) had chronic valvular degeneration, which may indicate choline and TMAO are worthy of further investigation.

### Methionine and cysteine

Sulfur-containing amino acids, methionine and cysteine, are used to synthesize taurine, a nonessential amino acid in dogs (Huxtable, 1992). However, many variables can affect the synthesis of taurine, such as lower bioavailable sources of amino acids, excess degradation during thermal processing, and shortage of methionine, which is a common dietary limiting amino acid (Mansilla et al., 2019). When methionine is sufficient, homocysteine is used to produced cysteine (Faeh et al., 2006). When methionine is deficient in the cell, homocysteine is re-methylated to form methionine (Harvey and Ferrier, 2011). The concentration ratio of S-Adenosylmethionine to S-Adenosylhomocysteine, substrates in the one-carbon metabolism pathway, defines the methylation potential of the cell. An increase in homocysteine, in the blood, decreases this ratio, leading to a decrease in methylation potential (Loscalzo and Handy, 2014). Additionally, the decreased ratio means an increase in the concentration of S-Adenosylhomocysteine. An increase in plasma S-Adenosylhomocysteine has been documented in patients with cardiovascular disease (Kerins et al., 2001).

Recent data have suggested that essential sulfur-containing amino acid requirements may be variable in different breeds and sizes of dogs (Mansilla et al., 2019). One study observed that large mixed breed dogs, averaging 38 kg in body weight, had a lower (50%) taurine biosynthesis rate than smaller dogs, averaging 13 kg, when fed a diet adequate in protein and sulfur-containing amino acids (Ko et al., 2007). Another study demonstrated that dogs eating diets containing ingredients, including animal meals, turkey, whole grain rice, rice bran, or barley, had low plasma methionine and cysteine concentrations (Delaney et al., 2003).

### Taurine

Taurine is a sulfur-containing amino acid that is not a component of proteins (Huxtable, 1992) and is synthesized endogenously in the liver of dogs from cysteine, which is catalyzed at the rate-limiting step, by the enzyme cysteine sulfinic acid decarboxylase (Huxtable, 1992). Taurine is biologically important for cardiovascular, skeletal muscle, and central nervous system function as well as the conjugation of bile acids in many mammals. Taurine increases intracellular concentrations of calcium ions, is an osmoregulator in the heart, and functions as an antioxidant in relation to cardiac function (Sisson et al., 2000). While taurine is not currently considered an essential amino acid in dogs, it is considered essential in cats. This is due to cats having a lower activity of cysteine sulfinic acid decarboxylase affecting the biosynthesis of taurine. Prior to supplementing taurine in cats’ diets, DCM was commonly diagnosed (Pion et al., 1990, 1998).

Studies evaluating if taurine should be considered an essential amino acid in dogs have not been conclusive. A study in 1998 observed that a diet, considered low in taurine for cats, fed to Beagles had no adverse effects (Pion et al., 1998). Another study showed that DCM related to decreased cysteine sulfinic acid decarboxylase and decreased dietary taurine in foxes (Moise et al., 1991). The results showed that foxes fed diets with 510 mg/kg of dietary taurine did not develop DCM while those from farms fed diets containing 300, 130, and 80 mg/kg had incidence of DCM. Some of the foxes from farms feeding diets with lower taurine concentrations did not have low plasma taurine or DCM, while others did. Foxes with postmortem examinations from the farm with a dietary taurine concentration of 300 mg/kg had significantly lower cysteine sulfinic acid decarboxylase activity than the foxes from the farm feeding a dietary taurine concentration of 510 mg/kg. This resulted in a renewed interest in heart disease related to the taurine deficiency in dogs.

Another study evaluated plasma taurine concentration in dogs with chronic mitral valve disease and dogs with DCM. Of the dogs diagnosed with DCM, 17% were found to have low taurine concentrations and the affected dogs were breeds that were not previously known to be predisposed to DCM, such as American Cocker Spaniels and Golden Retrievers. Despite these findings, the authors did not conclude that taurine was contributing to the development of DCM because predisposed breeds to DCM had normal taurine concentrations (Kramer et al., 1995). Conversely, a study referred to as the Multicenter Spaniel Trial (MUST) observed that Cocker Spaniels diagnosed with DCM had low plasma taurine concentrations, but when supplemented with taurine and l-carnitine had significant improvement in echocardiogram results within 4 mo (Kittleson et al., 1997; Sanderson, 2006). These improvements were significant enough that the dogs could discontinue cardiovascular pharmaceuticals.

The reason for the low plasma taurine concentrations in these dogs is unknown. However, it is speculated that a combination of an enzyme deficiency, such as cysteine sulfinic acid decarboxylase, and loss through conjugation with bile salts may be the cause. Taurine supplementation was warranted in these dogs since they had a documented taurine deficiency. Plasma total, free, and esterified carnitine concentrations were not low. However, this is a low indicator of myocardial carnitine concentration, and, prior to this study, two dogs that were given taurine supplementation without l-carnitine concentration did not improve. A few of the Cocker Spaniels had documented low myocardial carnitine concentrations; therefore, l-carnitine and taurine supplementation were given and were observed to provide the desired clinical and echocardiographic improvements (Kittleson et al., 1997). Moreover, the studies did not note if dogs were fasted or meal feed prior to sampling. However, a study in Labrador Retrievers evaluated the changes in plasma and whole blood taurine concentrations during 48 h of fasting and after feeding (Gray et al., 2016). Whole blood taurine status did not change during fasting; however, plasma taurine levels had a slight change but never decreased below normal ranges. Additionally, whole blood and plasma taurine concentrations were measured after a meal. While plasma taurine levels reached baseline 8 h after feeding, whole blood taurine did not fluctuate from baseline after a meal (Gray et al., 2016).

Currently, studies suggest that there may be breed-specific taurine concentration reference ranges and that one range cannot be applied across all breeds (Ko et al., 2007; Freeman et al., 2018; Kaplan et al., 2018, Ontiveros et al., 2020). Certain breeds are more associated with taurine-deficient DCM than others, as stated previously (Freeman et al., 2001; Backus et al., 2003). More recently, Golden Retrievers with taurine-deficiency related DCM have been studied (Belanger et al., 2005). It was reported that five related Golden Retrievers diagnosed with DCM showed improvements within 3-6 months after starting taurine supplementation. Additionally, recent studies (Kaplan et al., 2018; Ontiveros et al., 2020) have noted Golden Retrievers may be at risk for developing DCM but have failed to identify a definitive causal relationship between diet, taurine, and cardiac function. Specifically, the most recent study (Ontiveros et al., 2020) was not well-controlled from a nutritional standpoint. The study evaluated two groups: traditional diets and non-traditional diets; however, the non-traditional diet group was comprised of balanced and those used only for supplemental feeding, which are not formulated to meet AAFCO requirements. Furthermore, the majority of dogs eating the non-traditional diets did not have low taurine or abnormal cardiac parameters. Moreover, not all dogs with decreased systolic function had low taurine concentrations, and the relationship between whole blood taurine, plasma taurine, and cardiac muscle taurine concentrations remains unknown.

### Carnitine

Carnitine is a water-soluble molecule that is synthesized endogenously in the liver and the synthesis is regulated by exogenous supply (Neumann et al., 2007). Carnitine assists in the transport of long-chain fatty acids from the cytosol to the mitochondrial matrix; once inside, it undergoes beta-oxidation to generate energy (Stumpf et al., 1985; Mayes and Botham, 2003). Roughly, 60% of the total energy production for the heart is through beta-oxidation. Carnitine also plays an important role in the buffering of toxic levels of acyl CoA in the mitochondria to allow beta-oxidation to continue (Sugiyama et al., 1990). Therefore, deficiency in carnitine could cause cardiac dysfunction leading to cardiac diseases, including DCM (Keene et al., 1991; Mc Entee et al., 1995; Pion et al., 1998; Sanderson, 2006).

In the production of nutrients, including taurine and carnitine, there are several important cofactors and precursors, such as iron, (Ringseis et al., 2010), selenium, zinc, and niacin (Ko et al., 2007). The bioavailability of these nutrients is directly related to where they are located in the dietary matrix, which is known as a nutrient’s bio-accessibility (Holst and Williamson, 2008). For example, there are two forms of iron, heme and non-heme iron. If a large portion of the diet’s iron concentration came from non-heme iron sources, it is possible that this cofactor would be less bioavailable for the synthesis of carnitine. Heme iron is found in animal sources and is more readily available than non-heme iron, which is found in plants and animal sources. However, soybeans and unfermented soy can be a good iron source, if heme-iron sources are low. Polyphenols, found in red sorghum, legumes, spinach, betel leaves, apples, berries, citrus fruits, plums, broccoli, and oregano, can reduce non-heme iron and thiamine absorption. Another example is niacin, which is not readily bioavailable from mature maize (Gibson, 2007), and diets rich in maize could hinder carnitine biosynthesis.

Carnitine concentration can be assessed by three carnitine pools: the plasma, the myocardial, and the systemic, which is comprised of both (Borum, 1991). Low plasma carnitine concentration can help diagnose carnitine deficiency but may not correlate with myocardial concentrations (Borum, 1983). Plasma carnitine can have a normal or high concentration while myocardial carnitine can be low. Therefore, in order to accurately measure myocardial carnitine concentrations, endomyocardial biopsies can be performed (Pion et al., 1998).

Currently, only small case studies have reported a deficiency in carnitine associated with DCM in dogs. In an evaluation of three dogs at the University of Minnesota, all were found to be carnitine deficient, prior to the onset of DCM (Sanderson, 2006). Improvement was noted in a Miniature Dachshund given l-carnitine supplementation. Also, in a 1991 study, two Boxer littermates were diagnosed with DCM (Keene et al., 1991). One offspring had low plasma and myocardial carnitine concentrations at the time of diagnosis. Similarly, the littermate had low myocardial carnitine concentrations; however, plasma carnitine concentrations were within normal reference ranges. Regardless of plasma concentrations, both dogs were supplemented with l-carnitine and had a significant increase in fractional shortening (Keene et al., 1991). Another study reported two Boxers diagnosed with DCM (Costa and Labuc, 1994); one Boxer was supplemented with l-carnitine daily, while the other was never administered supplementation. Myocardial concentrations of carnitine only increased in the supplemented Boxer. Oral supplementation of l-carnitine, although increased fractional shortening and myocardial concentrations, did not mitigate DCM. However, this should be further investigated as a report of a Labrador Retriever, diagnosed with DCM and supplemented with l-carnitine, has shown increased concentrations of carnitine and improvements in electrocardiographic and echocardiographic indices (Mc Entee et al., 1995).

### Thiamine

Thiamine is an essential water-soluble vitamin in dogs. Thiamine exists in three forms; however, the most utilized and bioavailable form is thiamine pyrophosphate (Carpenter, 2012). Thiamine pyrophosphate plays a significant role in the metabolism of carbohydrates, lipids, and branched-chain amino acids (Depeint et al., 2006; Sriram et al., 2012; Manzetti et al., 2014; Abdou and Hazell, 2015). While most studies focus on thiamine deficiency and the adverse effects on the heart in pigs (Follis et al., 1943), rats (Suzuki, 1967; Bózner et al., 1969; Davies and Jennings, 1970), pigeons, mice, sea lions, foxes, and monkeys (Van Vleet and Ferrans, 1986), limited research is available in dogs. Reported adverse effects include dilated hearts in pigs (Follis et al., 1943) and cardiac mitochondrial alteration in rats (Suzuki, 1967; Bózner et al., 1969; Davies and Jennings, 1970). Although these studies gave insight into negative cardiac effects of thiamine deficiency, current research needs to be conducted to assess any potential connection to DCM in dogs. Another enzyme that can affect bioavailability is thiaminase. Thiaminase cleaves thiamine at the methylene linkage, rendering it biologically inactive. Uncooked fish, shellfish, Brussels sprouts, and red cabbage contain thiaminases (Gibson, 2007).

### Copper

Copper plays an important role in energy metabolism by assisting the function of critical enzymes (Harris, 2001). In addition, copper is needed in the production of hemoglobin, myelin, and melanin, as well as maintaining the strength of blood vessels, epithelial tissue, and connective tissue (Harris, 2001; Gropper and Smith, 2012). Thus, deficiency in copper can have detrimental implications for cardiac function (Klevay, 2000; Saari, 2000). Early studies have examined the effects of a copper-deficient diet on cardiac health in pigs, rats, and rabbits (Van Vleet and Ferrans, 1986), however, not in dogs. The effects of copper deficiency ranged from anemia, CHF, myocardial necrosis, calcification, and cardiac hypertrophy (Hunt and Carlton, 1965). Copper is routinely supplemented in complete and balanced canine diets and, therefore, absolute deficiency is rare. Regardless, research into relative copper deficiencies which could be caused by excessive dietary zinc or iron (NRC, 2006), and the correlation with potential copper deficiency and cardiac health in dogs, should be evaluated to rule this out as a contributing factor to the cause of DCM in dogs.

### Vitamin E and selenium

Vitamin E is a major lipid-soluble antioxidant present in plasma, erythrocytes, and tissues. Vitamin E works with the selenium-containing enzyme, glutathione peroxidase, to scavenge free radicals and prevent oxidative damage to polyunsaturated fatty acids (van Haaften et al., 2003). Dogs with DCM have significantly lower vitamin E concentrations and reduced glutathione peroxidase, which is involved in cysteine synthesis when compared with healthy dogs (Freeman et al., 2005). For example, a puppy diagnosed with cardiomyopathy exhibited signs of selenium-vitamin E deficiency and had distension of the left ventricle (van Rensburg and Venning, 1979). Furthermore, a study was recently conducted to observe the effects of selenium deficiency and protein-restricted diets on the myocardium in rats (Zhang et al., 2019). Increased concentrations of reactive oxygen species and malondialdehyde, which are measurements of oxidative stress, were observed. Additionally, along with a decrease in serum selenium, glutathione peroxidase activity was reduced.

Selenium bioavailability can also negatively affect the biosynthesis of taurine, by hindering the absorption of cofactors. Selenium is more available in its organic forms, seleno-cysteine and seleno-methionine, rather than the inorganic form, selenite. Overall, after processing nutrients from plants have improved the bioavailability due to the plant cell wall being compromised. However, animal sources have higher bioavailability properties after processing (Parada and Aguilera, 2007). Many biological activities of selenium occur through its incorporation into seleno-cysteine into seleno-proteins. Selenium deficiency is the main regulator of seleno-protein expression and has been associated with the pathogenicity of many viruses (Beck, 2007). Additionally, since selenium is a cofactor for glutathione, a deficiency in selenium leads to a decrease in glutathione peroxidase activity. In humans, selenium deficiency is associated with Keshan disease, an endemic cardiomyopathy that occurs in the areas of China with low soil selenium (Levander and Beck, 1997). Interestingly, selenium deficiency increases the occurrence of myocarditis and cardiomyopathy in mice exposed to both virulent and avirulent Coxsachie virus due to reduced glutathione peroxidase. Thus, glutathione peroxidase may protect against virally induced cardiac inflammation (Lubos et al., 2011) because reactive oxygen species may enhance viral replication (Beck, 2007).

### Internal factors and competitive and noncompetitive interactions

Internal factors, such as nutrient status of the animal, genetics, sex, pregnancy, lactation, chronic and acute infection, and disease state, can also affect the bioavailability of some nutrients (Gibson, 2007). Inter-nutrient competition also occurs when nutrients with chemical similarities compete for the same absorptive pathway, which is commonly seen when a supplement is taken with food. Also, noncompetitive interactions can occur. For example, phytates contained in unrefined cereals, legumes, nuts, and oilseeds will bind certain cations to form insoluble compounds (Williamson, 2017). This may lead to zinc, iron, calcium, and magnesium being poorly absorbed.

## Chemical-Inherent Compounds and Heavy Metals

### Cassava and cyanide

Other potential etiologies that could contribute to the development of DCM include chemical inherent compounds and heavy metals. A commonly used ingredient in pet food is cassava (tapioca), which contains 10% arginine (Crawford, 1968). Cassava is known to accumulate cyanogenic glycosides, cyanide. When cyanide is consumed, it is converted into thiocyanate, which requires sulfane sulfur from sulfur-containing amino acids (Tor-Agbidye et al., 1999). Thus, there is an increased demand for sulfur-containing amino acids during detoxification (Tor-Agbidye et al., 1999). This can limit the availability of sulfur-containing amino acids used to biosynthesize taurine and carnitine. Additionally, cassava has been identified as a goitrogenic food.

### Goitrogenic foods

Certain raw foods have been identified as goitrogenic, such as spinach, cassava, peanuts, soybeans, strawberries, sweet potatoes, peaches, pears, broccoli, Brussels sprouts, cabbage, canola, cauliflower, mustard greens, radishes, and rapeseed (Dolan et al., 2010). These foods have been observed to have properties that suppress the function of the thyroid gland, increasing the risk of hypothyroidism. This interferes with iodine absorption affecting growth, cognitive function, and hormonal balance. Additionally, hypothyroidism has been known to lead to DCM, more specifically hypothyroidism-induced canine DCM (Rastogi et al., 2018)

### Heavy metals

Ingredients used in pet food can contain heavy metals; however, the amount can vary depending on the mining, industrial processing, amount of pollution, and the usage of pesticide, fertilizer, and chemicals on the geographic location (Cao et al., 2010; Kim et al., 2018). For example, rice and certain leafy vegetables grown in China (Cao et al., 2010) along with cereal grains (Squadrone et al., 2017) are at a greater risk of accumulating heavy metals.

Heavy metals that are of major concern are arsenic, cadmium, and mercury. Since taurine detoxifies heavy metals, there is an increase in the demand, which may result in a taurine deficiency. More specifically, taurine depletion can occur during arsenic-induced cardiomyocyte viability, reactive oxygen species products, intracellular calcium, and apoptotic cell death (Flora et al., 2014). Taurine has also been observed to reduce cadmium-indices damages in murine hearts (Manna et al., 2008) and hypothalamus (Lafuente et al., 2005). A similar effect is exhibited in rats with taurine and its hepatoprotective effects against mercury toxicosis (Samipillai et al., 2010).

## Assessing Current Dietary Information

**Figure 2.**
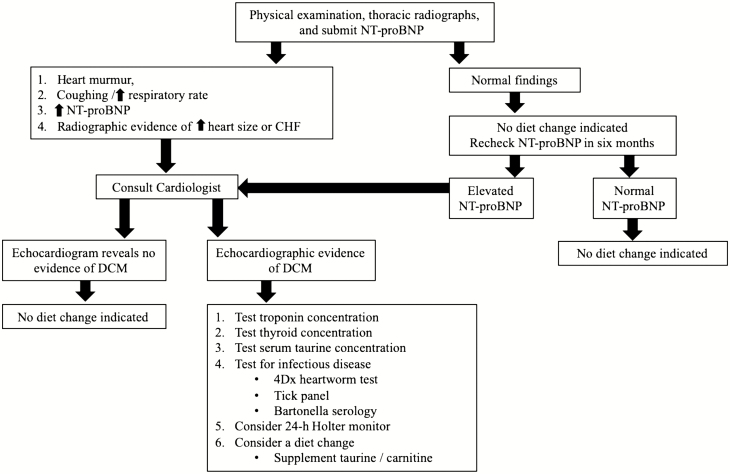
Recommended veterinarian guidelines for managing diet concerns for pets with and without clinical signs of cardiac disease.

The information above reveals that diet-associated DCM is not a new concern. However, recent reports of a potential association between diets with specific characteristics, such as, but not limited to, containing legumes, grain-free, novel protein sources and ingredients, and smaller manufactured brands have reignited an interest in this topic (Freeman et al., 2018). Despite no definitive correlation for grain-free diets or their ingredients to DCM, some veterinary cardiologists and researchers are recommending pet owners switch their dogs to grain-based diets, without exotic protein sources and avoiding boutique brands (NC State Veterinary Hospital, 2019). Yet, others state, there is insufficient evidence-based research on whether diet is the cause for the subjective claims (Freeman et al., 2018). With concerns for dietary inadequacies, a guideline has been proposed for veterinarians in diagnosing and managing DCM based on the multiple causes of DCM phenotype reviewed above (Figure 2).

## Assessing Current Limitations

A review of the current literature reveals gaps within DCM studies in dogs, including sampling bias, inconsistencies in sampling parameters, confounding variables, and lack of complete data for case studies on DCM and known genetic predisposition in certain dog breeds.

### Sample population

When conducting research, it is impossible to collect data from the entire target population; however, a subgroup, sample population, can be randomly selected. The importance of a sample population being randomly selected, without bias, is vital for researchers as they begin to extrapolate the information from the sample population to the target population (Banerjee and Chaudhury, 2010). In addition to a randomized, non-biased sample population, a power analysis should be conducted to estimate an appropriate sample size needed for meaningful data.

Major limitations can occur when extrapolating and generalizing data when the sample size is too small, biased, or has an overrepresentation of a subgroup (Pannucci and Wilkins, 2010). For example, one retrospective review comprised of dogs diagnosed with DCM from October 1997 to August 2001 (Fascetti et al., 2003) was limited in sample size. In this study, 64 dogs with DCM were initially evaluated. However, a reduction in the sample size occurred when confounding factors were accounted for. It was noted that 24, out of the 64 dogs, had blood samples submitted. Out of the 24, 14 had low blood or plasma taurine concentrations. From that subgroup of 14, 6 American Cocker Spaniels, a breed that is well-known to be predisposed to DCM, were excluded. An additional dog was excluded due to the lack of dietary history (Fascetti et al., 2003). However, Golden Retrievers and Newfoundlands, which are also high-risk breeds for DCM, were included (Fascetti et al., 2003; Freeman et al., 2018). In this small population of 12 taurine-deficient dogs, all were eating a commercial diet of lamb meal and/or rice as primary ingredients and 8 of these dogs were consuming the same diet. Due to the small sample size, this study’s data are difficult to extrapolate into the general dog population. Further studies to elucidate other potential causes of DCM in the excluded dogs that had normal taurine concentration are warranted.

Overrepresentation of a subset of a population can skew results. Recently, a study (Kaplan et al., 2018) collected taurine samples in 24 Golden Retrievers that were diagnosed with DCM and in 52 healthy Golden Retrievers. All Golden Retrievers with DCM were taurine deficient. Interestingly, some of the healthy dogs exhibited low taurine concentrations as well. This suggests that Golden Retrievers may represent breed sensitivity to DCM and the potential need for breed-specific taurine reference ranges (Kramer et al., 1995; Freeman et al., 2018; Kaplan et al., 2018). Due to the multifactorial etiology of DCM in Golden Retrievers (Kaplan et al., 2018), studies should not overrepresent them in the sample population if the goal of the study is to represent the dog population as a whole.

Since small sample sizes and overrepresentation of breeds are commonplace in DCM literature, the studies involving multiple breeds and larger sample groups are warranted to better understand if relationships exist between potential etiologies and the development of DCM for the overall dog population.

### Confounding factors and assessing independent variables

Confounding variables and lack of controlling for independent variables can introduce bias and suggest a correlation when none exists. For example, in 2018, a longitudinal study (Kaplan et al., 2018) was noted for having one of the largest sample size populations that assessed taurine concentration in Golden Retrievers. A significant improvement was noted in echocardiogram results and normalization of taurine concentrations when compared with baseline. This was observed after a diet change, administration of supplemental taurine, with or without l-carnitine, inotropic agents, diuretics, ACE inhibitors, and calcium channel blockers (Kaplan et al., 2018). Limitations listed were a lack of standardization among treatments, supplements and pharmaceuticals given, and diet changes. Additionally, the lack of medical records, including the duration on previous diet and use of different reference laboratories for analyzing blood parameters, adds to the confounding variables. Despite these inconsistencies, the study concluded that taurine supplementation could slow down the progression of DCM in dogs fed commercial diets. Since not all dogs received the same treatment, determining which variables had any correlation to these improvements can be challenging if not impossible. Future case studies should only include dogs with a complete dietary history and only one variable evaluated. Furthermore, variables that should be considered for future prospective research include age of subjects, body weight, body condition scores, litter sizes, birth weights, diet of the bitch, and diet of the puppy after weaning. These factors can be important as they may have the potential to impact the development of normal physiologic functions including cardiovascular health.

## Limitations to Current FDA Reports

From January 2014 to April 2019, 524 DCM cases were reported to the FDA (2019a). Reported cases have exponentially increased since the FDA’s initial communication to the public about a possible association between DCM and diets with specific characteristics, such as, but not limited to, containing legumes, grain-free, novel protein sources and ingredients, and smaller manufactured brands (Freeman et al., 2018). One case was reported in 2014 and 2015, two cases in 2016, and three cases in 2017. However, after the FDA’s initial release, 320 cases were reported in 2018, and so far, 197 cases as of April 2019 (Dodds, 2018; FDA, 2019a). These numbers included 515 dogs and 9 cats.

### Sample population

**Figure 3.**
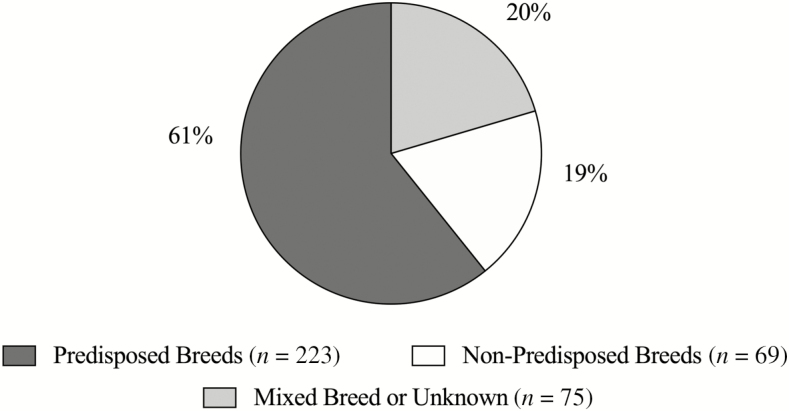
Percentage of dog cases reported in the FDA report that are breeds predisposed (dark gray) to DCM, non-predisposed (white) to DCM, and mixed or unknown breeds (light gray). Dogs considered predisposed to DCM include Great Danes, Doberman Pinschers, Boxers, Golden Retrievers, Cocker Spaniels, German Shepherds, and Bulldogs. FDA (2019a).

Recently, the FDA has stated that there is an increase in the prevalence of DCM in dogs. Based on the limited literature regarding DCM incidence rate, prior to the FDA reports, it is unknown whether this represents a true increase in cases. Most of the DCM cases reported to the FDA portal are a result of the FDA report on July 12, 2018, notifying the public of a potential link between DCM and pet food (FDA, 2019a). On June 27, 2019, the FDA released another update stating that 380 dogs were identified as “Breeds Most Frequently Reported to FDA” (Table 1). This information was from the cases reported up to April 30, 2019 (FDA, 2019b). However, the FDA report does not specify whether the dog breed categorization is based on breed phenotype or genotype. It is inferred that the term “breed” refers to phenotype in this instance because of the lack of genetic breed testing included in the FDA report. Thirteen of the dogs were reported “unknown breed”, 62 mixed breeds, and the remaining were named by breed. Of 305 dogs that had breeds listed (Figure 3), 223 (73%) were identified as a breed predisposed to DCM (Golden Retrievers, Great Danes, Doberman Pinschers, Boxers, Cocker Spaniels, and Bulldogs) (Calvert et al., 1997; Kittleson et al., 1997; Sisson et al., 2000; Broschk and Distl, 2005; Domanjko-Petric and Tomsic, 2008; Cunningham et al., 2018; Kaplan et al., 2018). Overrepresentation and breed reporting bias possibly inflated the number of reported cases (FDA, 2019a).

**Table 1.** Breeds of DCM cases most frequently reported to the FDA^1^

Breed	Number of cases
Golden Retriever	95
Mixed	62
Labrador Retriever	47
Great Dane	25
Pit Bull	23
German Shepherd	19
Doberman Pinscher	15
Australian Shepherd	13
Unknown	13
Boxer	11
Mastiff	8
German Shorthaired Pointer	7
Shetland Sheepdog	7
Weimaraner	7
American Bulldog	6
American Cocker Spaniel	6
Standard Poodle	6
Bulldog	5
Shih Tzu	5

^1^ FDA (2019a).

With the additional media around DCM, increased diagnosis of DCM may be seen due to the willingness of owners and veterinarians to investigate the disease. It is also possible that dog owners that are willing to invest in cardiology referral consultations are also more likely to have purchased implicated diets (FDA, 2019a). An increase in reports due to awareness or concern regarding diet history and veterinarians’ knowledge of predisposed breeds resulted in reporting bias and overrepresentation. For instance, the FDA report provided a detailed history of two Doberman Pinscher littermates, in the same household, eating a grain-free diet, and diagnosed with DCM via echocardiogram. Yet, these littermates are from a genetically predisposed breed, which is unlikely that diet-associated DCM is the primary, underlying etiology (Sammarco, 2008). To further understand the incidence rate and if there is a possible increase in DCM, multi-clinic, retrospective studies are warranted to identify a percentage of the population seeking referral to diagnose DCM, the incidence in specific breeds, and diet history, with comparison to market share.

### Sampling bias

The awareness of DCM and a possible association to the term, “BEG” diets, was raised in a veterinary medical publication (Freeman et al., 2018) and FDA reports (FDA, 2019a). “CVM [FDA’s Center for Veterinary Medicine] encourages veterinary professionals to report well-documented cases of DCM in dogs suspected to having a link to diet.” (FDA, 2019a) was quoted by the FDA in 2019. This demonstrates how asking for information in a certain way can skew data. Moreover, regardless of what diet the dog is eating, asking the veterinary community and the public for DCM cases in dogs only eating grain-free or exotic protein diets will result in sampling bias (Pannucci and Wilkins, 2010). To prevent this, the veterinary community should be asked to provide information for all DCM cases during the same time period, regardless of what the practitioners’ proposed etiology was.

From the documented cases, the FDA reports do not have complete medical records and diet histories for all dogs (FDA, 2019a). As of April 30, 2019, out of the 515 dogs, 14 dogs have provided initial samples for the 1 to 2-mo follow-up. Of these, only 10 dogs had information provided. In order to gain more information on these cases, Chesapeake Veterinary Cardiology Associates (CVCA) has been working to obtain medical records and follow up on FDA reported dogs (FDA, 2019a). Currently, for the 6-mo follow-up, the return rate is down by 50% due to incomplete sample collection or death of the dogs (FDA, 2019a). Ideally, inclusion criteria would be, at minimum, a diagnosis of DCM via echocardiogram, performed by a veterinary cardiologist and complete medical records and diet history, including the length of time on the diet. The information cannot be properly analyzed if a case report does not contain all required information; therefore, it should not be included in studies.

### Confounding variables

There were many confounding factors in the data collected from an FDA report where grain-free diets (or diets with pulses in the first five ingredients) were implicated as a potential cause of DCM. Potential confounding factors included incomplete diet history, age of the sample population, concurrent diseases, and unreported duration of the diet being fed at the time data were collected (FDA, 2019a). Another confounding variable is the body condition of these dogs and the number of treats and table scraps being given at home. Freeman et al. (2018) suggested that diet-associated DCM may be associated with treats rather than the actual diet being fed (Freeman et al., 2018). Additionally, the list of dogs was predominantly male, older than 80 yr of age, and no data on the concurrent disease were provided, as well as unreported duration of time on diet reported (FDA, 2019a).

**Figure 4.**
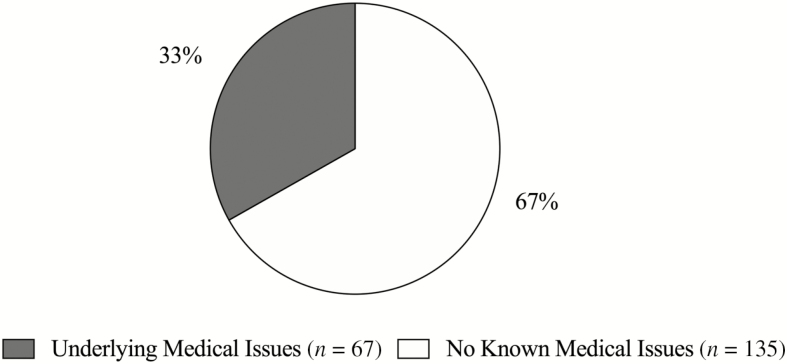
Dogs in FDA report diagnosed via echocardiogram that have known underlying medical conditions (gray) and dogs that had no known medical issues (white). Known underlying medical conditions include chronic valve disease, hypothyroidism, and tick-borne diseases. FDA (2019a).

The FDA Vet-LIRN report (FDA, 2019b) stated that 44% (*n* = 91) of the 202 dogs that had a diagnosis of DCM, confirmed by echocardiogram, also had documented concurrent medical conditions (Figure 4). These concurrent conditions can lead to the development of cardiovascular diseases. The report states 15% (*n* = 32) had valvular degeneration and 12% (*n* = 24) had atrial fibrillation. These conditions can contribute to an enlarged left atrium and ventricle and can resemble a DCM phenotype (Saunders and Gordon, 2015). The FDA Vet-LIRN report also stated 9% (*n* = 18) had concurrent hypothyroidism, which has been known to increase the risk of cardiovascular disease (Karlapudi et al., 2012). Lastly, 8% (*n* = 17) of the dogs had a concurrent diagnosis of tick-borne diseases, such as Lyme or Anaplasmosis. Studies indicate a possible relationship between Lyme disease and DCM in humans (Bartůnek et al., 2006; Kuchynka et al., 2015) and dogs (Agudelo et al., 2011).

The literature supports that 61% of dogs with chronic valvular disease or DCM have another concurrent condition (Freeman et al., 2003) leading to confounding factors when assessing DCM etiology. Therefore, dogs with these conditions, such as the cases mentioned previously, should not be included. Disease state should be considered when evaluating the results of the FDA investigation (FDA, 2019b). Future studies evaluating diet-association DCM should control for confounding concurrent medical conditions. Factors could include physical exam, blood nutrient concentrations, potential toxins, high sensitivity cTn1, NT proBNP testing, and echocardiograms.

### Standardized measurements

To decrease variability, standardized measurements are required. As previously discussed, the lack of standardization of treatments and data analyzing occurs in the studies related to DCM. These include the use of echocardiograms, whole blood vs. plasma taurine concentration, plasma vs. endomyocardial biopsy carnitine concentration, and not having definitive parameters for collecting diet histories. In recent FDA reports, of the 340 dogs’ medical records reviewed, 202 (36%) had a definitive diagnosis of DCM, confirmed by an echocardiogram (FDA, 2019b). The FDA Vet-LIRN states that 176 of the dogs diagnosed with cardiovascular disease had taurine measurements and echocardiograms (FDA, 2019b). However, the report does not provide details on whether taurine concentrations were from plasma, whole blood, or both. It has been suggested that whole blood taurine may be more indicative of the actual taurine status of the dog than plasma taurine (Freeman et al., 2018). This is likely from a higher concentration of taurine found in platelets and white blood cells than in extracellular fluid (Huxtable, 1992) Therefore, it may be difficult to compare data across various sample types. Future studies should be consistent across collections and analyses in order to give an accurate comparison.

### Conflicting information

**Figure 5.**
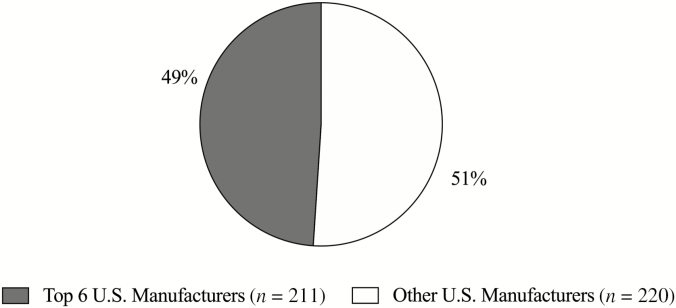
Top six U.S. manufacturers (gray) and other U.S. manufacturers (white) named in the FDA report concerning DCM in dogs. FDA (2019a).

Descriptors of pet foods implicated to have a subjective association with DCM are diets with specific characteristics, such as, but not limited to, containing legumes, grain-free, novel protein sources and ingredients, and smaller manufactured brands (Freeman et al., 2018). However, an exhaustive review of the literature provides evidence of conflicting information. For example, boutique diets, defined as produced by a small manufacturer, have been implicated in association with DCM (Freeman et al., 2018; FDA, 2019a). However, when the FDA report is broken down into which pet food manufacturers made the called-out diets (FDA, 2019a), 49% of the brands listed were made by one of the six largest pet food manufacturers in North America (Petfood Industry, 2019). Given that almost half of the brands listed on the FDA report (FDA, 2019a) on June 27, 2019, are not manufactured by boutique pet food companies (Figure 5), it is unlikely that an association can be made to DCM.

A bar graph in the 2019 FDA report titled, “Animal Proteins in Diets Reported to FDA,” lists 630 reported protein sources. The FDA figure lists the top seven proteins of the implicated diets: chicken, lamb, salmon, whitefish, turkey, beef, and pork. These comprised 76% of the diets named in the FDA report (FDA, 2019a), which are not exotic pet food proteins (Case et al., 2011). Previously, based on a study examining 12 dogs (Fascetti et al., 2003), lamb meal-based diets were implicated in taurine-deficient DCM. These ingredients are inherently low in taurine and dogs fed these diets had low whole blood taurine concentration (Delaney et al., 2003). In addition, lower plasma methionine and cystine concentrations were identified in animals fed meals containing turkey (Delaney et al., 2003). Further, suggesting exotic protein sources may not lead to the development of DCM.

The current hypothesis coming from data presented to the FDA is that grain-free diets could have an association with the development of DCM (Freeman et al., 2018; Adin et al., 2019), despite the lack of support from the literature. For instance, a study concluded that whole blood taurine concentrations were lower in dogs fed whole grain diets, such as rice bran and barley (Delaney et al., 2003), while another study observed that beet pulp, rather than rice, had a greater impact on lowering the concentrations of plasma and whole blood taurine (Ko and Fascetti, 2016). This suggests that these ingredients are likely not a cause of the taurine-deficiency in dogs. However, the FDA has currently raised concern for diets that contain legumes as one of the top seven ingredients.

According to the FDA’s 2018 report (FDA, 2018), lentils, peas, and other legumes (pulse ingredients) have been speculated to be responsible for diet-associated DCM. However, this hypothesis may be unsupported by evidence-based research. In a study conducted at the University of Illinois, in a controlled environment, dogs were fed 45% legumes or fed a diet primarily comprised of poultry byproduct. Interestingly, over the 90-d study, there was no significant difference when comparing plasma amino acids between groups (Reilly and de Godoy, 2019). Additionally, comparing the plasma and whole blood taurine concentrations between groups was not significantly different (Reilly and de Godoy, 2019). Therefore, since there was no change in taurine concentrations with dogs being fed a diet containing 45% legumes, legumes are more than likely not a cause of taurine-deficient DCM. Further supporting this, Kaplan et al. (2018) compared commercial diets being consumed by dogs diagnosed with DCM (Kaplan et al., 2018). While all initial ingredients in the ingredient list were not reported, this study did report 12 out of 13 diets were grain-free and 10 out of 13 contained legumes in the first five ingredients. However, 22 out of 23 of these dogs were eating less than the maintenance energy requirements and brand suggested feeding guidelines. This may have resulted in a suboptimal intake of sulfur-containing amino acids (Kaplan et al., 2018) and not pulse ingredients. This exhaustive review of the literature provides support that eliminates the pet food characteristics that have been implicated to have a subjective association with DCM. To determine if DCM can be associated with certain categories of diets would require further prospective studies that remove confounding factors.

## Conclusion

Recently, a correlation between diets with specific characteristics, such as, but not limited to, containing legumes, grain-free, novel protein sources and ingredients, and smaller manufactured brands to DCM has come under scrutiny by academic researchers and the FDA. The use of the acronym “BEG” and its association with DCM are without merit because there is no definitive evidence in the literature. At this time, information distributed to the veterinary community and the general public has been abbreviated synopses of case studies, with multiple variables and treatments, incomplete medical information, and conflicting medical data and opinions from veterinary nutrition influencers. Also, in past literature, sampling bias, overrepresentation of subgroups, and confounding variables in the data weaken this hypothesis. Additionally, based on current literature, the incidence of DCM in the overall dog population is estimated to be between 0.5% and 1.3% in the United States. However, the FDA case numbers (560 dogs) are well below the estimated prevalence. Therefore, it is impossible to draw any definitive conclusions, in these cases, linking specific diets or specific ingredients to DCM.

DCM is a multifactorial medical condition with many proven etiologies and potential causes contributing to the development of the disease. Therefore, prospective studies investigating, not only diet, but also infection, metabolism, and genetic involvement, must be conducted. In hopes of better understanding a potential correlation with diets to DCM, more objective data need to be collected and analyzed, without sampling bias and confounding factors. While determining the cause of recently reported cases of cardiac disease is of the utmost importance, based on this review of the current literature, there is no definitive relationship these implicated diet characteristics and DCM.

## Abbreviations

AAFCO: The Association of American Feed Control Officials
ACE: angiotensin-converting enzyme
BEG: boutique manufacturers, exotic proteins, and grain-free diets
CHF: congestive heart failure
cTnI: cardiac troponin-I
DCM: dilated cardiomyopathy
FDA: Food and Drug Administration
ECG: electrocardiogram
NT-proBNP: N-terminal end of brain natriuretic peptide
SCD: sudden cardiac death
TMAO: trimethylamine N-oxide
