# Corrigendum to: Review of canine dilated cardiomyopathy in the wake of diet-associated concerns

**DOI:** 10.1093/jas/skaa209

**Published:** 2020-07-10

**Authors:** Sydney R McCauley, Stephanie D Clark, Bradley W Quest, Renee M Streeter, Eva M Oxford

**Affiliations:** 1 BSM Partners, Bentonville, AR; 2 The Heart Vet, Ithaca, NY

Corrigendum to: “Review of canine dilated cardiomyopathy in the wake of diet-associated concerns” by Sydney R. McCauley et al. *Journal of Animal Science* 2020; doi: 10.1093/jas/skaa155.

In response to reader inquiries we are adding the following statement for clarity: BSM Partners provides nutritional advice for pet food companies of all sizes, including both those that produce grain-free, pulse-rich pet foods, and those that produce grain-inclusive, pulse-free pet foods.

